# The first recorded outbreak of epidemic dropsy, 1877–80: Climate, empire, and colonial medical science between India, Bengal, and Mauritius

**DOI:** 10.1017/mdh.2024.24

**Published:** 2024-10

**Authors:** Yadhav Deerpaul, Alexander Springer, Philip Gooding

**Affiliations:** 1 Department of History, University of Wisconsin-Madison, Madison, WI, USA; 2 Indian Ocean World Centre, McGill University, Montreal, QC, Canada

**Keywords:** Epidemic Dropsy, Mauritius, India, Colonialism, Climate, Colonial Science

## Abstract

This article reconstructs the first outbreak of epidemic dropsy recorded in documentary evidence, which occurred in Calcutta, Mauritius, and northeastern India and Bengal in 1877–80. It uses current medical knowledge and investigations into the wider historical contexts in which the epidemic occurred to re-read the colonial medical literature of the period. It shows that colonial policies and structures in the context of variable enviro-climatic conditions increased the likelihood that an epidemic would break out, while also increasing the vulnerability of certain populations to infection and mortality. Additionally, it shows how the trans-regional nature of the epidemic contributed to varying understandings of the disease between two colonial medical establishments, which influenced each other in contradictory ways. The article’s core contributions are to recent trans-regional perspectives on disease transmission and colonial medical knowledge production in the Indian Ocean World.

## Introduction

This article reconstructs the first outbreak of epidemic dropsy recorded in documentary evidence. This epidemic occurred in 1877–80 in Calcutta and Mauritius, with additional cases being reported in other parts of colonial Assam and Bengal. It was spread by the consumption of a contaminated foodstuff and recorded by colonial medical officers in India, Bengal, and Mauritius in the *Indian Medical Gazette* and other medical publications. Re-reading this medical archive – considering modern scientific knowledge and historical investigations into the wider environmental, social, and political conditions within which the epidemic occurred – sheds new light on its origins, spread, and effects. Further, it allows for an investigation into the multiple influences on late-nineteenth-century colonial medical knowledge production, including across the Indian Ocean between South Asia and the Mascarene Islands. The article shows the importance of a trans-regional perspective for understanding disease transmission and medical knowledge production in the Indian Ocean World during the era of European colonialism.^1^

The period in which the epidemic occurred has received significant attention in the environmental history of the Indian Ocean World, a macro-region stretching from eastern Africa to eastern and southeastern Asia. The year of the outbreak coincided with possibly the largest positive El Niño Southern Oscillation (ENSO)^2^ and Indian Ocean Dipole (IOD)^3^ anomalies of the last 250 years.^4^ This contributed to extreme rainfall across the Indian Ocean World: severe droughts occurred in southeastern Asia, northern China, parts of the Indian subcontinent, and southeastern Africa, and excessive rainfall occurred in parts of equatorial eastern Africa.^5^ As Mike Davis has shown, the severity of the drought coupled with exploitative colonial grain and labour policies contributed to widespread famine in the Bombay and Madras Presidencies of colonial India.^6^ Diseases were also widespread in these contexts, notably with epidemics of cholera, smallpox, and a range of famine-related diseases, including beriberi, occurring in and across several locales.^7^

Calcutta, wider Bengal, and Mauritius are understudied in this global environmental and epidemiological context. This may partly be because rainfall and harvests were regular throughout the period in Bengal. Mauritius, meanwhile, experienced above-average rainfall during the summer rainfall season of 1877, which probably contributed to increased transmission of malaria in and around sugar plantations.^8^ There were not massive floods that burst the banks of rivers, however, as was the case on parts of the African mainland opposite Zanzibar.^9^ Nevertheless, as discussed below, the wider environmental context, especially the drought in southern India, played a significant role in the origin and transmission of epidemic dropsy between Bengal and Mauritius. Understanding the epidemic in these terms builds on the framing of the Indian Ocean World as an interconnected space, including through climatic teleconnections, environmental conditions, and disease transmission.^10^

Key factors that affected the outbreak of epidemic dropsy in 1877–78 include the colonial response to El Niño-related drought in southern India, which contributed to the inflation of food prices in Calcutta and wider Bengal; the spread of other epidemics in colonial India and Bengal, notably cholera, smallpox, and malaria; and the economic structure of colonial Mauritius, which was built on the labour of Indian indentured migrants as monocrop sugar producers. These factors increased the likelihood that epidemic dropsy would both break out in Calcutta and then spread to Mauritius. Moreover, they probably increased the vulnerability of the suburban poor around Calcutta to mortality after infection, and they increased the chances of transmission to (and mortality of) a certain group of indentured labourers in Mauritius, namely those who originated in Bihar. How the first recorded outbreak of epidemic dropsy emerged and then spread was very much tied to the colonial and environmental contexts of the period.

Of course, the colonial medical establishments of both India and Mauritius were oblivious to most of these factors. Instead, in the late nineteenth century, they largely understood epidemic dropsy through the medical challenges that their respective colonies were already facing. These included attempts in India to understand other tropical diseases that they had yet to fully comprehend and attempts in Mauritius to minimise the effects of epidemics spreading from India. However, investigations into epidemic dropsy also shed light on how medical knowledge was shared and negotiated between different colonies. Colonial medical practitioners in both India and Mauritius used each other’s studies to inform their understanding of the disease. They did so, however, in contradictory ways, with different practitioners using the same reports from different colonies to support conflicting arguments. This thread of the article supports recent contributions to the medical history of the Indian Ocean World that emphasise the multiple spatial and contextual origins of modern medical science.^11^

There are limitations to this analysis, however. Reliance on colonial medical documents means that the interpretations of local populations are often obscured. Local ‘voices’ rarely come through in the primary source material consulted for this article, except in the form of case reports, which say little about the contexts within which they were made. Memoirs or newspaper reports could be key here, and the present authors would welcome work that brought in such evidence. This could be especially important for thinking about medical knowledge production. It is argued here that a trans-regional perspective between different medical establishments is essential for understanding colonial medical science. Evidence from local sources may further suggest how colonial medical practitioners incorporated evidence from the societies within which they lived. Such work would build upon that of many others’ approaches since the 1990s, which have pointed out how ‘western’ science, including medical science, was influenced by numerous local contexts and knowledge bases, including from South Asia.^12^

The remainder of the article is divided into three substantive sections and a conclusion. The first section outlines current medical knowledge about epidemic dropsy and uses it to assess the first recorded epidemic’s origins and spread in India, especially Calcutta, in 1877–80. It assesses the wider environmental, economic, and epidemiological contexts that made the city and certain people more vulnerable to infection and mortality. The second section discusses the spread of epidemic dropsy to Mauritius in 1878–79. It shows that the island colony’s labour policies and economic structure increased the chances of the epidemic spreading among certain demographic groups, contributing to heightened mortality amongst indentured labourers from Bihar. The final section examines the colonial medical response to the epidemic in both India and Mauritius. It argues for a trans-regional approach to understanding colonial knowledge production, showing how India-based medical practitioners attempted to incorporate reports from Mauritius to support their scientific arguments, despite doing so in contradictory ways.

## Epidemic dropsy, its origins, and its spread in India and Bengal

After an individual is infected, the first symptoms of epidemic dropsy usually appear after five days. They include diarrhoea and vomiting, which lasts between a few days and a week. Other early symptoms can include fever, lower backache, and hair loss. Subsequently, between around nine days and two weeks, oedemas form at the lower end of the limbs. They are regularly painful, burning, and itching and become worse upon the infected individual standing. Other related symptoms can include anaemia, palpitations, and breathlessness. In extreme cases, it can cause heart failure, pneumonia, respiratory distress syndrome, and renal failure. The mortality of untreated infected people is usually around 5%.^13^ The first confirmed epidemic occurred in Calcutta in 1877–80, although unconfirmed reports suggest it may have been present on a French ship carrying indentured migrants in 1863 and in northeastern India since the 1866 Orissa famine, which also affected Bengal.^14^ Since then and up to the end of the twentieth century, outbreaks of epidemic dropsy were a regular occurrence in different parts of India, most recently around New Delhi in 1998.^15^ Epidemics are also known to have occurred in Myanmar, Fiji, and Mauritius.^16^

The cause of epidemic dropsy, which was confirmed in the 1940s, is the ingestion of edible oils that have been adulterated with Mexican poppy (*Argemone Mexicana*) oil.^17^ Specifically, it is caused by two toxic alkaloids, sanguinarine and dihydro-sanguinarine, contained within Mexican poppy seeds, which are used to make oil. The Mexican poppy originates in Central America but it became naturalised throughout the tropics following European expansion across the Atlantic from the late fifteenth century. It was probably brought to the Indian subcontinent via Portuguese, Dutch, and/or English ships. By the late nineteenth century, it was prevalent throughout Bengal and across northern and southern India.^18^ It grew (and continues to grow) wild and favours sandy and rocky soil, including on roadsides and in dried riverbeds. In the nineteenth century, there were debates within India’s colonial medical and botanical establishment about Mexican poppy oil’s uses, particularly in terms of its possible medicinal and/or narcotic qualities. Several sources also indicate its usage in local medicinal practice, and at least one source from Cuttack, Orissa noted that it was ‘mixed with mustard seed as an adulteration’.^19^

Undoubtedly, this mixing of Mexican poppy and mustard seeds/oils led to the 1877–80 epidemic. Mustard oil was and remains the premier cooking oil in South Asian cuisine, especially in Bihar and Bengal. However, it is regularly difficult for both vendors and consumers to differentiate adulterated from unadulterated mustard oil. The seeds and oils of both plants are similar, with the seeds being small, round, and dark and the oils a clear yellow. Thus, accidental adulteration is possible, especially when mustard and Mexican poppies are grown in proximity.^20^ Mustard plants (*Brassicaceae*) are also less hardy to water stress than Mexican poppies, and they are susceptible to several diseases and pests. Therefore, the care and environmental conditions required to ensure a good crop may have incentivised producers and/or vendors to adulterate their mustard oil with the oil of the weed-like Mexican poppy, as the latter is regularly more available. Indeed, drought and its effects on agriculture during the 1866 Orissa famine may tentatively be suggested as a trigger for adulteration becoming more widespread. This hypothesis was made more possible for the late nineteenth century when the link between using Mexican poppy oil as an adulteration and epidemic dropsy was unknown.

The 1877 outbreak almost certainly originated in Bengal. This statement contradicts that of David Arnold, who suggested in 2010 that it first broke out in the Madras Presidency.^21^ His argument was based on the 1886 report of Norman Chevers.^22^ However, Chevers was one of several colonial medical officers in India who, until the 1930s, argued that epidemic dropsy was a form of beriberi. The latter disease is caused by thiamine (vitamin B1) deficiency, and its symptoms regularly include oedemas similar to that of epidemic dropsy. Beriberi was rife in the Madras Presidency in 1876–78 due to the famine conditions caused by disastrous colonial grain and labour policies in the context of severe El Niño-related drought.^23^ There is no known evidence (apart from that provided by colonial medical officers who conflated it with beriberi)^24^ that epidemic dropsy caused by ingestion of adulterated cooking oil broke out in the Madras Presidency in these years, even if drought and starvation probably limited the harvest of mustard seeds. Additionally, even in years of regular climate, Calcutta did not regularly import mustard oil from the Madras Presidency. Rather, Calcutta’s supply came from the ‘brown mustard’ (*Brassica Juncea*) grown in rural Bengal and Bihar, supplemented by other varieties and rapeseed oil brought via the East Indian Railway.^25^ Thus, Arnold’s suggestion based on some colonial medical officers’ reports that epidemic dropsy broke out first in the Madras Presidency is probably a result of misdiagnosing cases of beriberi and other famine-related diseases.

Nevertheless, the wider enviro-climatic conditions affecting much of the rest of colonial India increased the likelihood of an outbreak in Calcutta in 1877. Despite regular rainfall and agricultural production in Bengal in 1876–78, colonial responses to dearth in wider India, especially in the Madras and Bombay Presidencies, contributed to the price of staples doubling in Calcutta between 1875 and 1877.^26^ Additionally, epidemiological conditions in the city were generally adverse, with epidemics of cholera, malaria, and smallpox all breaking out between 1876 and 1880. According to colonial statistics, there were 7,253 deaths from cholera in Calcutta in 1877–80, as opposed to 1,693 in the four years preceding (and 958 of those were in the first six months of 1873). The 1877–80 cholera epidemic also spread to wider Bengal, especially along the Ganges and Brahmaputra rivers and the East Indian Railway.^27^ These economic, social, and epidemiological conditions heightened levels of desperation, especially in the poorer suburbs. Even with what was likely a regular harvest of mustard seeds in rural areas, they provided an ample incentive to adulterate mustard oil with Mexican poppy oil, as doing so extended the supply of cooking oil and reduced the need to purchase more at inflated prices.

After the initial outbreak in 1877, the epidemic made its appearance every year until 1880. Its prevalence, however, was not continuous. Rather, cases and deaths rose and declined according to the seasonal cycle. According to Kenneth McLeod, a colonial medical officer who was present in Calcutta at the time of the epidemic, it broke out every year in 1877–79 towards the beginning of the cooler, dry season (October–April). Its earliest recorded occurrence in any year was in 1878, on 4 September. Thereafter, cases almost ceased entirely at the onset of the warmer, monsoon season (May–September).^28^ Although McLeod did not make the connection, this pattern aligns with the agricultural season for mustard grown in Bengal and elsewhere in northeastern India.^29^ Mustard is sown in Bengal in September–October and is harvested in January–March, depending on the sub-region and strain.^30^ Thus, fresh mustard seeds and oil tended to be in their shortest supply towards the end of the southwest monsoon season. Some evidence from wider colonial Bengal suggests that the price of mustard oil could nearly double from the beginning of the monsoon season to the drier season.^31^ Adulterating it with Mexican poppy oil by the latter time, especially in the context of higher prices and the desperation that characterised late-1870s Calcutta, could have been a strategy used by producers and/or vendors to try to keep prices down.

**Figure 1.**
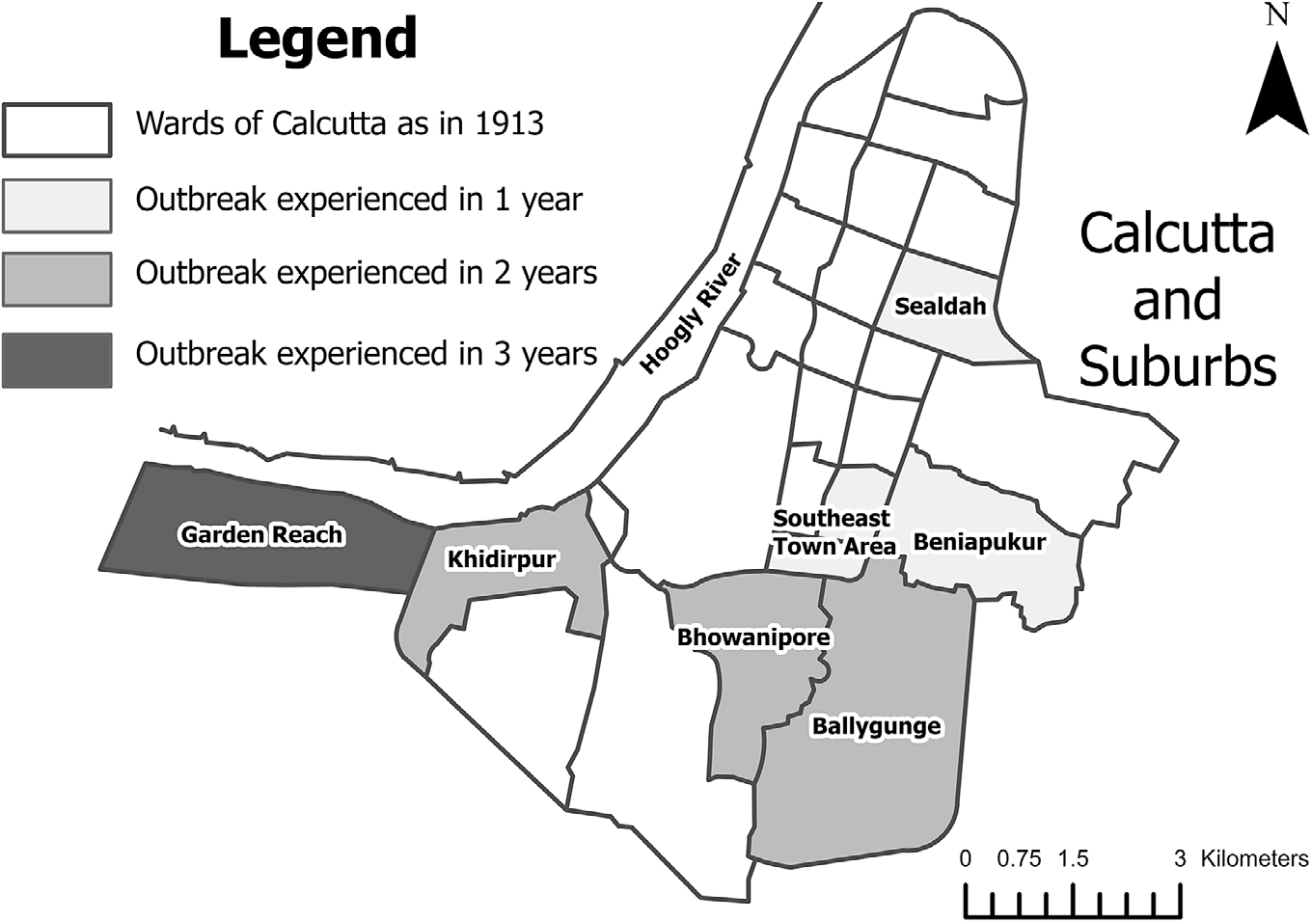
Map of Calcutta and suburbs with areas with reported infections highlighted according to year. Digitised map based on E.P Richards, ‘City of Calcutta’ (1913): https://curiosity.lib.harvard.edu/scanned-maps/catalog/44-990114901360203941 [accessed: 12 June 2024].

The spatial distribution of cases of epidemic dropsy also varied from year to year, except that it was most prevalent in Calcutta’s poorer suburban areas throughout the epidemic (see Figure 1). In 1877–78, it was largely confined to the southern suburbs in and around Garden Reach, on the eastern bank of the Hooghly River. Being the location of the port, this was a major transit area for goods and people travelling between the hinterland, the city, and the wider world. In 1878–79, it broke out in the same places and spread to neighbouring districts, namely Khidirpur, Bhowanipore, and Ballygunge. Finally, in 1879–80, it broke out across Calcutta’s southern suburbs, as well as (for the first time) in parts of the southeastern town area and the eastern suburbs of Beniapukur and Sealdah. It was also present in Shillong and some other locales in Assam in 1878–79 and 1879–80, as well as in Dhaka, eastern Bengal (present-day Bangladesh) in 1879–80.^32^ These patterns suggest that the practice of adulterating mustard oil with Mexican poppy oil was limited to only a few suppliers/vendors in specific locales. However, it became more widespread as high prices took their toll over time. The end of the epidemic followed the re-establishment pre-famine food prices in 1879–80.^33^

Notwithstanding these broad trends, it is difficult to assess the actual numbers of cases and deaths from epidemic dropsy in colonial Bengal and India during these years. As mentioned, many among the colonial medical establishment were slow to identify epidemic dropsy as a singular disease, conflating it with other diseases, especially beriberi, instead.^34^ According to McLeod, meanwhile, colonial medical practitioners in Calcutta regularly ascribed deaths from what he believed to be epidemic dropsy to ‘fever’, thus mixing statistics for epidemic dropsy with those of malaria (which was also epidemic in Calcutta at the time) and other diseases.^35^ McLeod also pointed to a small increase in the number of deaths attributed to dropsy (as opposed to epidemic dropsy), a generic disease that can be caused by phenomena, including malnutrition, other than consumption of adulterated cooking oil.^36^ Some of these cases may also have been beriberi, especially as some famine-affected individuals travelled from the Madras Presidency to Calcutta in search of food and/or wages.^37^ Thus, McLeod was probably correct in his assessment that statistics for ‘dropsy’ probably did not reflect the severity of epidemic dropsy in Calcutta in these years.^38^ There are thus no official tabulations of cases and deaths from epidemic dropsy in Calcutta (like there are for, for example, cholera), however flawed such official tabulations regularly were.

Even so, some measure of the spatial severity of the epidemic is still gleanable from colonial sources. Using police reports, McLeod suggested a very high death rate in and around Calcutta: about 20% in the town area and 44% in the suburbs. He also estimated a 19% death rate from his own investigations in nearby villages.^39^ The recorded death rate was much lower outside of Calcutta and its environs, with apparently only a total of three people succumbing in Shillong.^40^ This is probably reflective of two phenomena. First, the recorded death rate for Calcutta is probably inflated. Most cases, especially milder ones, were probably not reported or treated where either the police or McLeod could count them. Also, both counts probably included other diseases that were epidemic in Calcutta during the late 1870s, including malaria.^41^ Second, despite these qualifications, the death rate in Calcutta was still probably higher than it was elsewhere in Bengal and northeastern India. Unsanitary conditions, the effects of other diseases on the immune system, and diminished diets caused by inflation probably reduced infected individuals’ capacities to recover, especially among the poor.^42^ The specific socio-economic conditions that arose from colonial responses to El Niño-related drought heightened the chances of an outbreak of epidemic dropsy in Calcutta in 1877, and they may have increased the vulnerability of some suburban populations to death after infection.

## The spread of epidemic dropsy to Mauritius in 1878–79

Epidemic dropsy broke out in Mauritius in 1878–79. Indentured labourers who had come to Mauritius from Bihar via Calcutta were recorded as being the most affected population. The system of indenture was instituted in 1834 following the abolition of slavery in the British Empire and lasted until the 1910s. Although often brutal and having many of the same characteristics of plantation slavery, indenture was partly distinguishable from slavery because it was cemented with a written labour contract.^43^ During the indenture period, just under 500,000 labourers arrived in Mauritius from colonial India and Bengal, largely to maintain and expand the monocrop sugar economy. In 1878–79, 5,216 indentured migrants made the journey.^44^ Of those, 3,092 departed from Calcutta (2,262 in 1878 and 830 in 1879).^45^ Disease has been a common theme of historical analysis in this context, notably with case studies focusing on the spread of cholera in 1819 and 1854–56 and malaria in 1864–67 from India.^46^ Such analyses refer to the spread of disease on ships and within Mauritius via human-to-human transmission and the spread of vectors, notably Anopheles mosquitos. This section furthers this historiography by examining another way in which disease spread between these two British colonies: the trans-shipment of food, specifically the trans-shipment of adulterated mustard oil.

Since the cholera and malaria epidemics of the 1850s and 1860s, the colonial medical establishments of India and Mauritius were highly conscious of the dangers of disease transmission via the indenture system. Arrival of infected migrants and infection of established labourers were bad for business. Thus, each colonial government instituted measures to limit the occurrence of trans-colonial epidemics. Recruits in India underwent a medical examination by the civil surgeon before the signing of the indenture contract and before embarkation.^47^ There were also quarantine stations on Mauritius’ outer islands, including at Gabriel Island and Flat Island, as well as at Pointe aux Canonniers, if cases of epidemic disease were identified aboard ship.^48^ Then, once ships arrived in Port Louis, the Depot Medical Officer vaccinated unprotected migrants from smallpox and made further checks for outbreaks of other diseases.^49^ It was only after these several rounds of checks that newly arrived indentured migrants were taken to sugar estates.^50^ The success of these measures was such that, in 1877–80, neither cholera nor smallpox broke out in Mauritius, despite them being epidemic in Calcutta at that time. Colonial Mauritius’ Depot Medical Officer reported ‘no epidemic sickness’ brought via new arrivals of indentured labourers in 1879.^51^

Of course, this does not discount the possibility that at least some of the just over 3,000 arrivals from Calcutta in 1878–79 were infected with epidemic dropsy before they arrived in Mauritius. They boarded ships in Bhowanipore, one of the epicentres of the epidemic.^52^ Thus, given the incubation period between infection and the manifestation of symptoms, some may have become infected in Calcutta and still passed the pre-embarkation medical examination. Moreover, meals for indentured migrants aboard ships destined for the sugar colonies, including Mauritius, were cooked with mustard oil or ghee.^53^ If the mustard oil was acquired near the ships’ departure point in 1878, then there is a reasonable chance that it was adulterated with Mexican poppy oil, which could have led to an outbreak on board. However, little evidence suggests that epidemic dropsy was prevalent on journeys between Calcutta and Mauritius in 1878–79. One informant to a colonial physician in Mauritius indicated the presence of ‘some people with enflamed legs’ aboard one ship, but this was an exception. Another report directly contradicted the hypothesis of large numbers of infections on board.^54^ Uncertainty in this context is enhanced by their rarely being medical officers aboard ships of indentured migrants.^55^ Moreover, at the same time as denying the presence of ‘epidemic sickness’ among new arrivals, the Depot Medical Officer also noted the presence of what we now know to be epidemic dropsy on sugar estates.^56^ This discrepancy in reporting suggests that the absence of ‘epidemic sickness’ in the Depot Medical Officer’s report cannot be attributed to him simply missing significant numbers of infected individuals as they passed through the port; he was clearly able to recognise an unusual disease. Thus, the number of infected individuals arriving in Mauritius was probably minimal, at least compared to the number of people who were eventually infected.^57^

Instead, the spread of epidemic dropsy in Mauritius can be attributed to the nature of the island colony’s food supply during the late nineteenth century. The centrality of sugar production to Mauritius’ colonial enterprise disincentivised domestic food production, making the island reliant on food imports, including cooking oil. Thus, ships of indentured migrants from Indian ports were also laden with food, which was sold to Port Louis’ shops and wholesale to the depot.^58^ Indeed, this dependency on imports was beginning to grow at the time of epidemic dropsy’s outbreak owing to what is known as the *grand morcellement* from about 1875. While it had smaller-scale antecedents in previous decades that had given rise to small cane farming, the *grand morcellement* signified the dividing and selling of large sugar estate lands to several smaller landholders, especially to former indentured labourers who had fulfilled the terms of their contract and had adopted Mauritius as their home.^59^ Such small landholders had to focus on sugar production to make their enterprises profitable, meaning that small tracts on the edges of large sugar estates that may once have been put towards domestic food production were increasingly given over to sugar.^60^ This is despite references to the presence of mustard on the island.^61^ Economic conditions encouraged (former) indentured labourers to purchase the ‘cheapest articles’ from estate shops.^62^ Dependency on food imports from India in the context of the latter’s socio-economic challenges in the late 1870s increased the chances of Mauritius’ exposure to adulterated mustard oil.^63^

The hypothesis that most infections of epidemic dropsy occurred in Mauritius rather than pre-embarkation or aboard ship is also supported by a critical examination of the numbers of infections reported by the island colony’s medical establishment. Andrew Davidson, a colonial medical officer, suggested that between 80,000 and 100,000 people, or a quarter of the entire island’s population, were infected at some point,^64^ although this is probably an overestimation. McLeod later estimated 20–30,000 infections.^65^ Additionally, 729 deaths were registered in Mauritius as ‘acute anaemic dropsy’ – which we now know to be epidemic dropsy – in 1879.^66^ If this number is generally acceptable and the mortality rate was around 5% (as has been the case with most outbreaks of epidemic dropsy since the late nineteenth century), then this would put the number of infections at under 15,000. The addition of some numbers from 1878 and accounting for infections not observed by the colonial medical establishment as well as for misdiagnoses of other diseases would still produce a figure closer to the lower end of McLeod’s estimate.^67^ Even so, these numbers dwarf the figure of just over 3,000 new arrivals from Calcutta in 1878–79. This strongly indicates that most of those infected in Mauritius had been present in the island colony since before the disease broke out.

**Figure 2.**
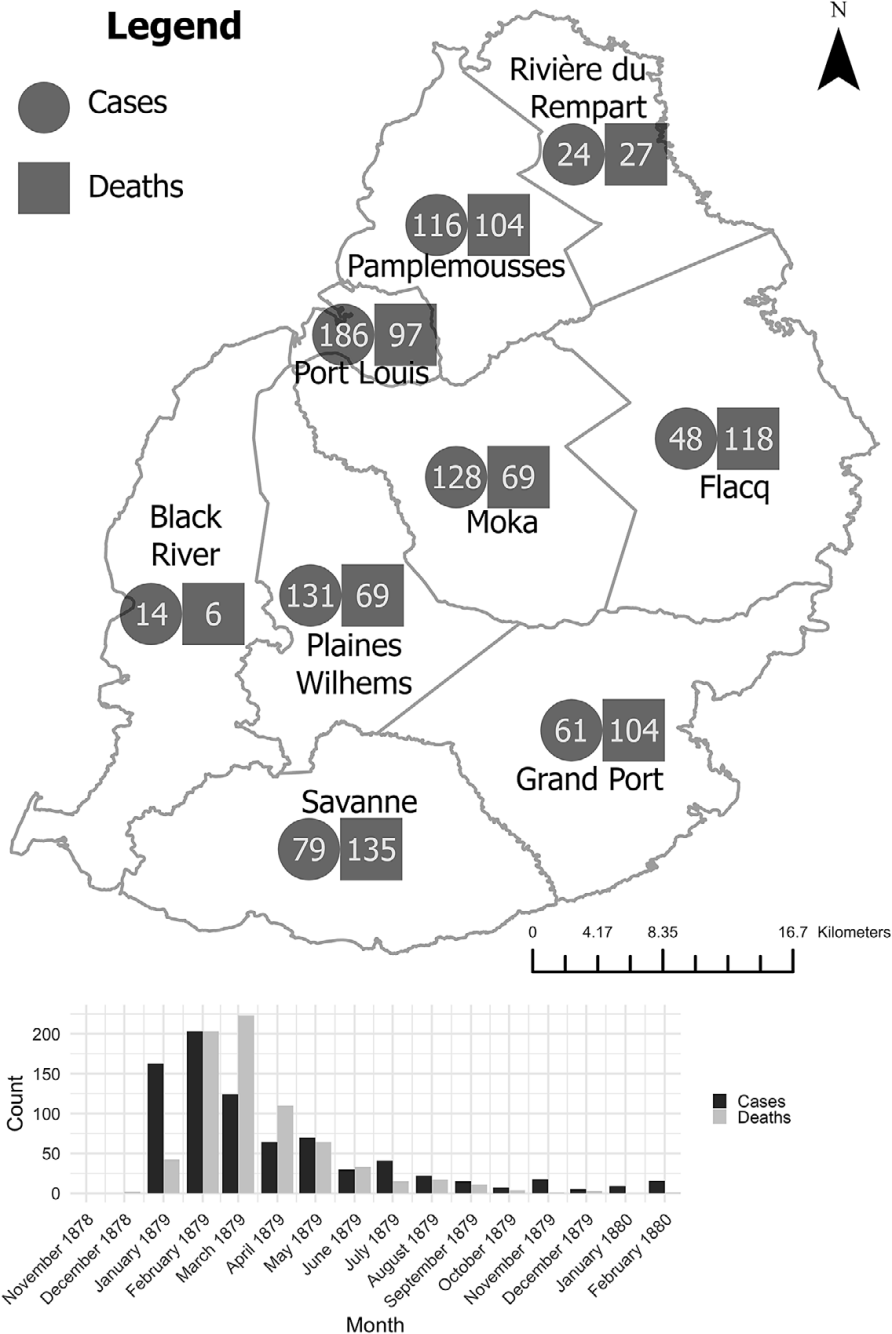
Reported cases of beriberi, acute dropsy, acute anaemic dropsy, anasarca, and other diseases’ mortality from November 1878 to February 1880. Data taken from: Francis Lovell, ‘Report on Acute Anaemic Dropsy in Mauritius’, *Indian Medical Gazette*, 16, 12 (1881), 343–4 [Return C and Table D].

Further, colonial medical reports indicate that the disease first broke out in Moka, on the Central Plateau.^68^ Since the 1860s, when drought and disease pushed many planters from littoral zones, this was part of the island colony’s core sugar-producing area.^69^ One medical officer identified a ‘peculiar’ form of dropsy in November 1878 in this locale, while also suggesting that it may have been present as early as September or October.^70^ This broadly aligns with the chronology of the outbreak in Calcutta in that year, with the first official report in the latter also dating from September, possibly suggesting that they were linked to the same batch of adulterated mustard oil.^71^ The disease then spread, by December 1878, to nearby Plaines Wilhem and Port Louis, the colonial (and current) capital on the western coast. Subsequently, from January 1879, infections enveloped most of the island’s littoral regions, including Savanne, Pamplemousses, Flacq, Riviere du Rempart, and Black River. The epidemic reached its greatest extent in February, when it also appeared in Grand Port, on the southeast of the island, before it receded from April and disappeared entirely by July.^72^ Thus, the travel of infection probably went from inland to the coast, rather than the other way round (see Figure 2).

**Figure 3.**
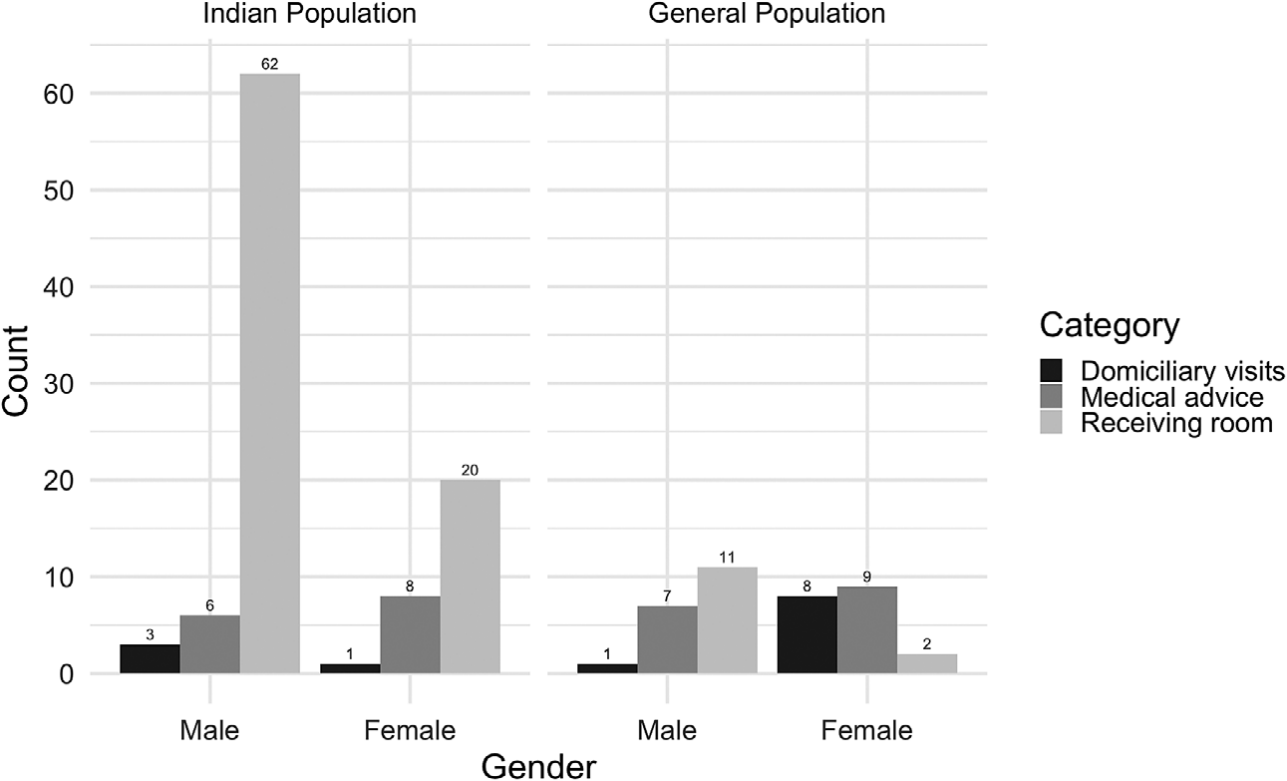
Return of acute dropsy cases from January to March 1879. Data: Lovell, ‘Report on Acute Anaemic Dropsy’, (1881), 346 [Return].

Colonial medical sources indicate that indentured labourers who originally boarded ships in Calcutta suffered the highest rate of infection throughout the epidemic. Those who travelled from Madras were apparently also severely affected, though not to the same degree.^73^ By contrast, it only spread among the wider populace in Port Louis, a cosmopolitan centre where a wider demographic would inadvertently have had access to the supply of adulterated mustard oil.^74^ Reports from colonial hospitals also indicate that more men were infected than women (see Figure 3). Nevertheless, it is necessary to be careful with such reports. There is a possibility, for example, that colonial medical officers who drew parallels between the symptoms of the novel disease in Mauritius and Calcutta may have overestimated infections amongst indentured labourers from Calcutta to emphasise their scientific case. Certainly, some believed (wrongly) for a time that new arrivals from Calcutta spread it to others already based in Mauritius via human-to-human transmission.^75^ Nevertheless, even with these qualifications, this rough outline about the extent to which it affected different groups may be broadly acceptable because of what is known about the indenture system in the late 1870s.

Most indentured labourers who boarded ships in Calcutta originated in Bihar. They arrived in the port city via the Ganges and Brahmaputra rivers and the East Indian Railway. Since the end of the 1850s, the annual number of new arrivals had decreased precipitously. The peak figure of 44,397 Indian migrants, of which 23,180 were from Calcutta, in 1859, for example, dwarfs those of the late 1870s.^76^ By this time, rather than being replaced with new arrivals, indentured labourers increasingly renewed their contracts or purchased small tracts of land on which to grow their own sugar.^77^ The relatively few new arrivals, meanwhile, were largely recruited by ‘returnees’ – those who had finished their contracts in Mauritius and were employed to return to Bihar to recruit more to follow in their footsteps. Returnees were encouraged to recruit people from their kin group, as well as entire families. This process was officially sanctioned by the Mauritian colonial government and was more effective in the hinterland of Calcutta than around Madras and Bombay. It was viewed as more cost-effective than using local contractors, and it promised to provide a longer-term solution to the labour question, as whole families might be more likely to stay in Mauritius after their initial contract than individuals, who may have wished to return to their kin network in Bihar.^78^

The result was that, by the late 1870s, the labour camps that served the sugar estates and were populated by indentured labourers who boarded ship in Calcutta were fairly insular social places. Richard B. Allen refers to a ‘social structural stability’ within the camps. This social structure was governed by the concepts of family and village, both of which were influenced by prior connections to Bihar.^79^ The camps of labourers comprised close-knit communities of around 300 individuals.^80^ These conditions provided a fertile environment for epidemic dropsy to spread amongst particular demographic groups, especially those who originated in Bihar because of the relative success of the ‘returnee’ system from that locale. Further, the high proportion of reported male versus female infections suggests that most were infected via consumption of mustard oil in rations provided by sugar estates to plantation workers, who were mostly male.^81^ However, lower visibility of female-dominated domestic spaces to colonial observers may mean that several infections of women were overlooked.^82^ Harsh working conditions on the plantations may additionally have made male sugar workers more vulnerable to the disease’s more severe symptoms, thus increasing their chances of mortality and that they would be noted in colonial medical documents.^83^ In any case, the social structure of labour camps within the context of the wider indenture system may have made labourers who originally boarded ships in Calcutta more vulnerable than other groups to infection with epidemic dropsy in the late 1870s.

The spread of epidemic dropsy to and within Mauritius in 1878–79, therefore, was contingent on a range of local and global factors, both in the short and long terms. Heightened reliance on food imports from India made the island colony more vulnerable to exposure to certain food-borne diseases, such as epidemic dropsy, especially in the context of drought, famine, and inflation in India and Bengal during the late 1870s. This history stands in contrast to other types of trans-oceanic disease transmission to Mauritius at this time, such as that of cholera and smallpox via human-to-human contact, whose longer-term influence had contributed to the implementation of several preventative measures. Further, the social and organisational structures that underpinned sugar production and the indenture system by the late 1870s may have contributed to certain groups in Mauritius – namely indentured labourers who originally boarded ships in Calcutta – being more susceptible to infection with and mortality from epidemic dropsy. Vulnerability to disease and its effects in Mauritius were in some ways built into the imperial structures that linked the island colony to India and Bengal during the nineteenth century.

## Colonial medical understandings and responses

The outbreak of epidemic dropsy in the late 1870s elicited a significant reaction from the colonial medical establishments of India, Bengal, and Mauritius. Speaking at the third meeting of the Calcutta Medical Society in March 1880, Dr. E.W. Chambers, a medical officer based in Calcutta, said, ‘Nothing [had] been of more interest to the [medical] profession within the [previous] few months.’^84^ Much of this interest was theorised and discussed in the *Indian Medical Gazette*, a monthly medical journal founded in 1866 as a forum for colonial medical practitioners to exchange and improve knowledge about diseases in India. Within its pages, there are a multitude of reports on what we now know to be epidemic dropsy from Calcutta, Shillong, Dhaka, and Mauritius dated between 1878 and 1882. McCleod then published the culmination of the knowledge gained from the 1877–80 epidemic, and the debates therein, in 1893.^85^ Close analysis of the publications in this context sheds significant light on the nature and circulation of trans-oceanic, intra-imperial medical knowledge in the late nineteenth century. It also shows how a much smaller medical establishment in Mauritius came to leave an outsized imprint on colonial medical knowledge production in India. This history builds on recent contributions to medical history in the colonial Indian Ocean World, which emphasise the multiple spatial and contextual origins of ‘modern’ medical science.^86^

As is well known, colonial medical knowledge was constrained by several scientific and institutional factors in the late nineteenth century. Miasma theory, which attributed the spread of disease to noxious air, still predominated, and germ theory remained a minority position, especially amongst colonial medical practitioners. Interpretations of miasma theory were compatible with racialised tropes rooted in environmental determinism.^87^ The reports on epidemic dropsy contained in the *Indian Medical Gazette* are filled with postulations about the cause of the disease, and they regularly explore race and climate as possible triggers.^88^ Some reports do, however, mention the possible role of specific foods, but even these were couched behind an inherent racism. For example, Andrew Davidson, a colonial physician based in Mauritius, argued that diets based on rice ‘predisposed’ people to some of epidemic dropsy’s symptoms, specifically anaemia.^89^ Such discourse was rooted in a colonial belief system that associated rice cultures with negative racial stereotypes.^90^ None of these reports examined the possibility that a specific ingredient in the common diets of the island became adulterated or compromised at a particular time. This only came later, notably with an outbreak of epidemic dropsy in Fiji via mustard oil imported from India in the 1920s.^91^

Understandings of epidemic dropsy in the late 1870s were also rooted in the specific contexts within which the respective medical establishments of India and Mauritius worked during the late 1870s. This meant partially understanding the outbreak in terms of the diseases or challenges that they were already familiar with or were still investigating. In India, one early report suggested that what we now know to be epidemic dropsy was a ‘malarious fever’, while also linking it to malnutrition.^92^ But the overriding paradigm through which they sought to understand the outbreak was through identifying it with beriberi.^93^ The first report in the *Indian Medical Gazette* to give the disease a name, which is dated to January 1879, used the title, ‘Acute Dropsy (Beriberi?)’, despite earlier reports suggesting it was a ‘new disease’.^94^ Others titled their papers similarly soon after, and in April–May 1880, one prominent medical practitioner discussed the outbreak in Calcutta simply under the heading ‘Beri-beri’ while drawing direct comparisons with the outbreak of said disease in the Madras Presidency.^95^ The influence of this line of thinking was such that by May 1881, it had become the prevailing opinion among the colonial medical establishment in India and Bengal that what we now know was epidemic dropsy was ‘the same disease as has been described by observers in Madras and Ceylon under the term Beri-beri [sic]’.^96^

The pervasiveness of the conflation of epidemic dropsy with beriberi was partly rooted in struggles within India’s colonial medical establishment to understand beriberi itself. Indeed, around this time, India’s colonial medical officers were also conflating beriberi with other diseases, such as kala-azar (visceral leishmaniasis).^97^ Broadly speaking, until the 1870s, they understood the disease as a distinctly Indian problem, despite outbreaks and accompanying medical literatures in and from other parts of the tropical world.^98^ The outbreak of epidemic dropsy in Calcutta in 1877–80 and their linking it to beriberi in the Madras Presidency, meanwhile, allowed them to classify beriberi as an ‘epidemic fever’. Their doing so served two purposes. First, it enabled them to demonise and criminalise internal migrants as ‘fugitives’, especially supposedly infected individuals from Madras who some claimed spread it to Calcutta.^99^ Second, according to Arnold, it ‘seemed to give their investigations into beriberi a unique insight and an international mission that, in the competitive world of tropical medicine, might put British India on a par with, even ahead of, investigations elsewhere’.^100^ The idea that beriberi could be epidemic had the potential to be regarded as an important medical discovery, with consequences for the medical profession across the tropical world. The conflation of epidemic dropsy with beriberi in India owed much to political circumstances within India and to the colony’s medical establishment’s position in the production of global medical science.

The influence of the beriberi paradigm for understanding the epidemic in Calcutta was such that it affected early reporting in India about the outbreak in Mauritius in 1878–79. The first editorial of India-based medical practitioners in this context, dated August 1880, was entitled ‘Beri-beri in the Mauritius’.^101^ The article sought to answer two questions: First, whether the disease was imported into Mauritius from Calcutta, and second, if it was beriberi. In response to the first question, the authors were cautious, noting that the ways they understood the evidence (based on comparable symptoms, demographics of those infected, and the epicentres of the epidemic) were largely circumstantial. Such caution contrasted, of course, with the concurrent rhetoric perpetuated by some medical officers that linked the epidemic in Calcutta to the migration of ‘fugitives’ from the Madras Presidency, which was based on similarly limited evidence.^102^ In this context, there may have been an initial reluctance to acknowledge that new epidemics originating in India, such as the one under review, could travel internationally, as Michael Christopher Low has observed in relation to cholera around the same time. Acknowledging international communicability had the capacity to provoke new measures, such as enhanced quarantine restrictions, that might limit British India’s mercantile fleet, which was integral to its prosperity. Such prosperity was of much higher priority than public health to British India, including to its medical establishment.^103^

In response to the second question, about whether the outbreak in Mauritius was beriberi, the authors were equivocal. Apart from the problem of possibly having to acknowledge the disease, which they had up to that point understood as a distinctly Indian problem, could travel from India to other regions, their response was complicated by the observations of colonial medical officers in Mauritius itself. In this context, they drew on a report by Francis Lovell, colonial Mauritius’ Chief Medical Officer. The report was dated 28 April 1880 and was briefly summarised in the same edition of the *Indian Medical Gazette* as the editorial.^104^ It was not published in full, however, until December 1881–January 1882, a delay that may be indicative of the controversy arising from it.^105^ Rather than in terms of beriberi, Lovell’s report referred to the disease as ‘acute anaemic dropsy’, arguing that there were significant differences between the two diseases. It also used the term ‘epidemic dropsy’ in text, which is probably the first written usage of the term.^106^ At the same time, while challenging the beriberi thesis, Lovell shed doubt on the veracity of investigations into beriberi in India as a whole by stating that the disease had only been ‘loosely defined’.^107^ Despite perpetuating the ‘beriberi paradigm’ in headlines, doubts stemming from geo-political factors and interpretations from Mauritius started to creep into some of the colonial Indian medical establishment’s discourse by mid-1880.

**Figure 4.**
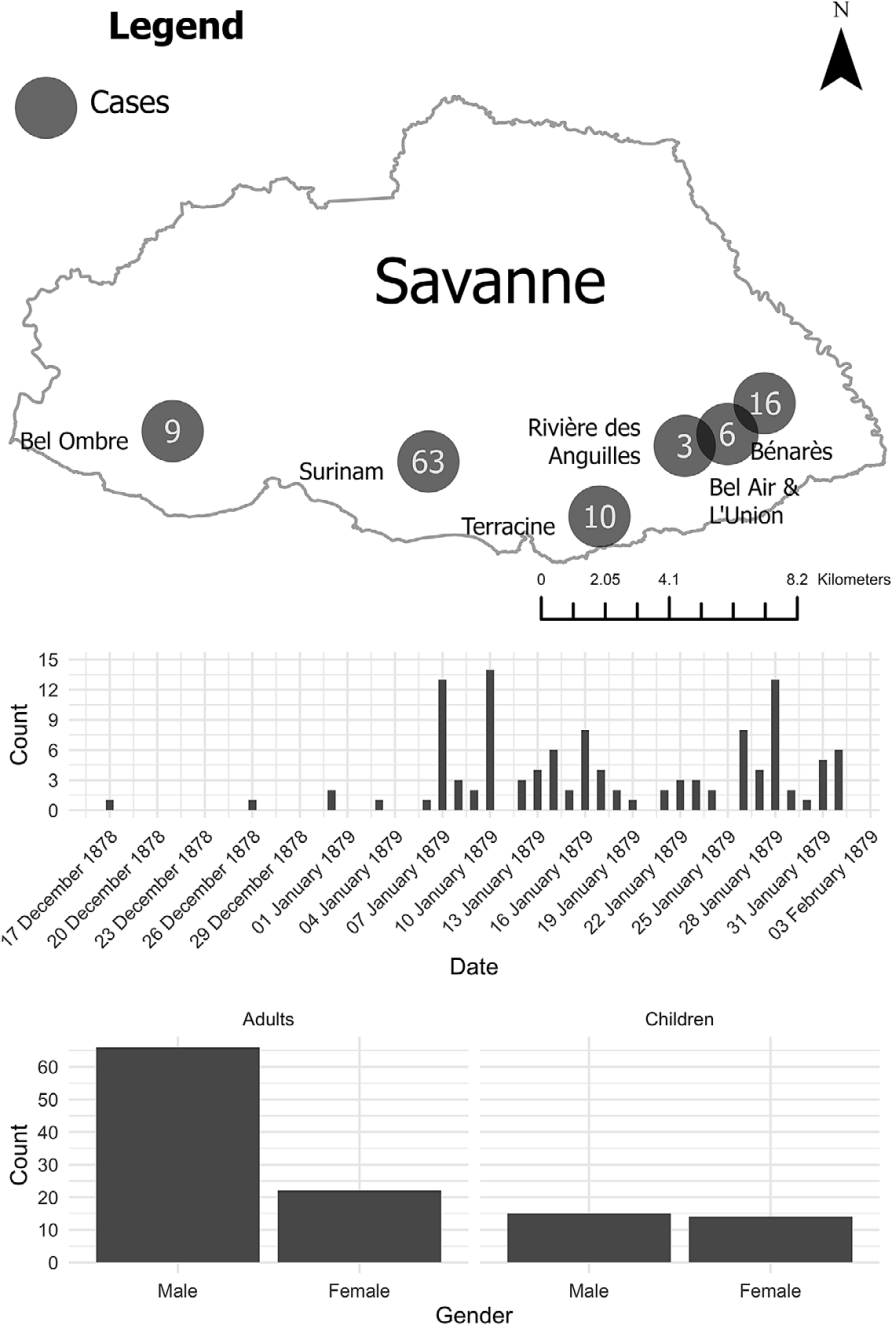
Reported cases of ‘acute anaemic dropsy’ on estates in Savanne from 17 December 1878 to 1 February 1879. The tables detail the daily progression of epidemic dropsy in six sugar estates and the gender and age distribution. The tables do not contain the precise year, but such has been inferred from descriptions and previous tables. Data: Lovell, ‘Report on Acute Anaemic Dropsy,’ (1881), 346–7 [Table E].

**Figure 5.**
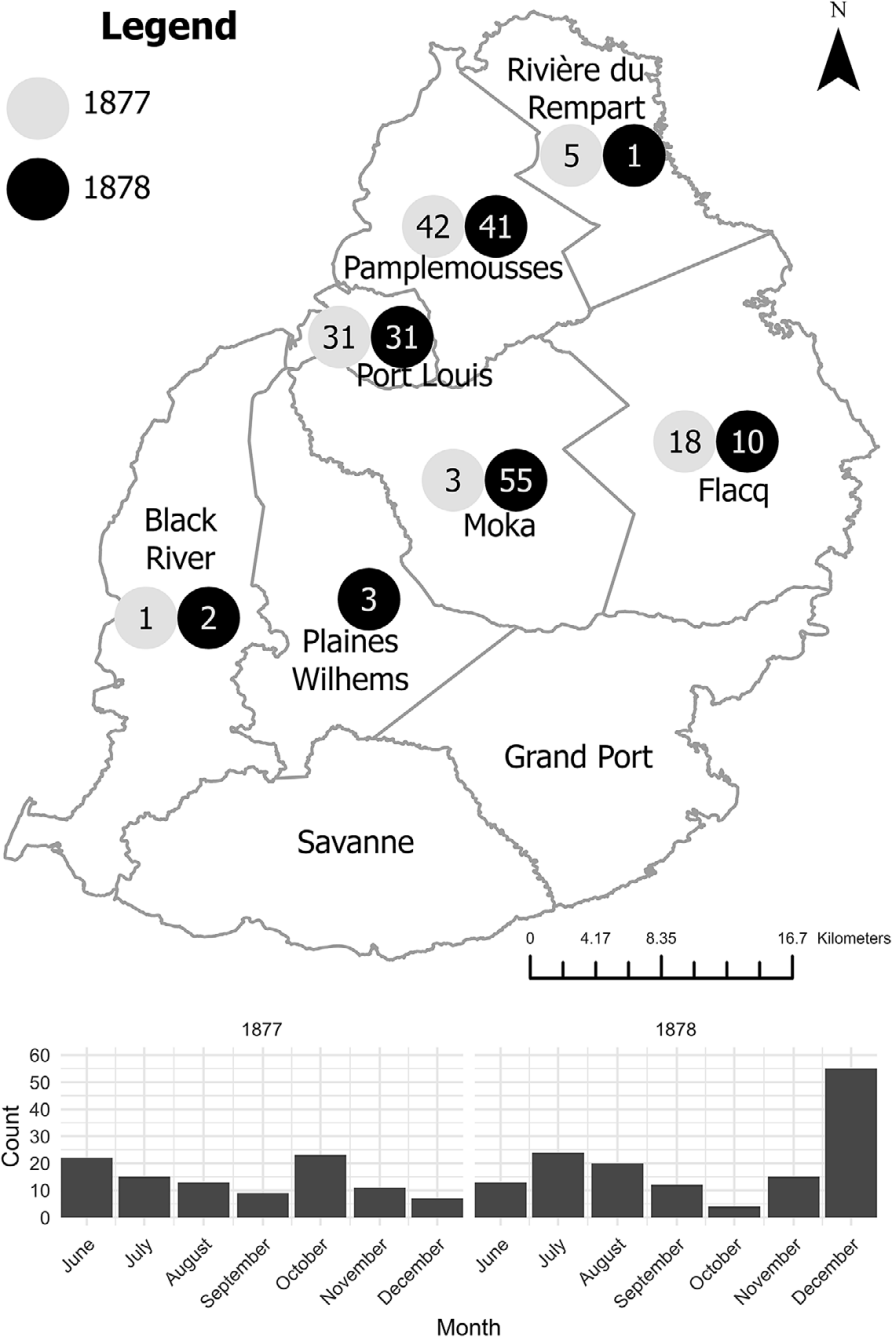
Reported cases of anasarca, oedema, and dropsy cases from June to December 1877–1878. While Dr Clarenc learned of a peculiar disease distinct from ‘dropsy, anasarca and oedema’ with symptoms similar to beriberi in November 1878, Lovell reinforced the theory that the disease was present before, and he compiled both the 1877 and 1878 tables to show such trends. The December peak in 1878 was attributed mainly to the district of Moka, with 45 cases as the disease became ‘general’ there. Table A makes mention of ‘Total Mortality’ in the final row, but this was probably a typographical mistake, as there is no mention of mortality in the associated paragraph of Lovell’s report, and the corresponding Table B does not make mention of mortality. Lovell, ‘Report on Acute Anaemic Dropsy’, (1881), 342–3 [Tables A and B]. Map based on: A. Descubes, ‘Map of the Island of Mauritius’ (1880): https://nla.gov.au/nla.obj-231272435/view [Accessed: 27 July 2023].

It is probably unsurprising, therefore, that medical knowledge about epidemic dropsy developed along vastly different lines in Mauritius than in India. As the April 1880 editorial in the *Indian Medical Gazette* stated, ‘The Mauritius cases [were] under more close and systematic medical observation than those which were seen in Calcutta.’^108^ Colonial physicians in Mauritius interviewed patients and tracked hospital admissions, and they attempted to tabulate numbers of deaths from the disease at district and (sometimes) estate levels (see Figure 4).^109^ Further, Lovell used regular reports on the transmission of – and mortality from – a range of diseases that bore similar symptoms to what we now know was epidemic dropsy, such as anasarca and generic dropsies and oedemas, across several years (see Figure 5). Beriberi, not being present in Mauritius at this time, was not one of them. This enabled him to dismiss early suggestions that epidemic dropsy was related to malaria or was a form of anasarca, and to ignore the Depot Medical Officer, who relied on information from India to refer to the epidemic as beriberi.^110^ Instead, he was able to use colonial physicians’ in-country fieldwork to strongly suggest that what he called ‘acute anaemic dropsy’ arrived in Mauritius in September–October 1878, despite the first formal observation only being written in November of that year.^111^ The comparatively small colonial medical establishment in Mauritius mobilised its resources in ways that suggested scientific rigour, enabling a challenge to mainstream colonial medical thinking in India.

Mauritius’ colonial medical establishment’s capacities in this context were rooted in the island colony’s labour and economic structures. As Yoshina Hurgobin has argued, ‘the colonial state viewed the worker’s body as a crucial link to the regime of production processes since workers’ health directly determined his or her production.’^112^ This was no truer than in Mauritius, where the colonial economy was dependent on indentured workers’ production of sugar and therefore also on workers’ health. Maintaining workers’ health in this context, however, was complicated by the colonial apparatus’ unwillingness to invest capital in measures that would enhance sanitary conditions, improve workers’ diets, or provide adequate hospitals.^113^ Indeed, the existence of estate hospitals in Mauritius since the 1830s has largely been understood in terms of colonial control rather than health – indentured labourers were only admitted once their condition was desperate.^114^ The death rate at said hospitals was consequently shockingly high.^115^ Thus, the colonial medical establishment saw scientific investigations that identified diseases as the more cost-effective strategy for understanding, and therefore limiting, the spread of epidemics. Medical science was understood as fundamental to Mauritius’ sugar economy.

This self-imposed mandate for Mauritius’ colonial medical establishment, which linked workers’ production and the health of the economy to advancements in medical science, was especially strong in the late 1870s. Although epidemics of cholera that spread from ships of indentured labourers had taken a massive toll in the 1810s and 1850s, the arrival of malaria in the mid-1860s provided longer-term challenges. Unlike cholera, which could be periodically eradicated through quarantine measures, malaria continued to spread – colonial medical science had yet to link the disease to Anopheles mosquitos, instead relying largely on miasma theory.^116^ Moreover, it was particularly virulent during seasons or years of above-average rainfall, as damper hydrological conditions are conducive to mosquito reproduction and expansion of their territory, especially on sugar plantations.^117^ The transmission and death rate spiked in 1867 and 1877, both of which were years of above-average summer rainfall triggered by positive ENSO and (in 1877) IOD anomalies.^118^ Thus, epidemic dropsy broke out in 1878–79 during a period of heightened concern among Mauritius’ medical establishment about the effects and spread of disease among the island colony’s indentured labourers.

The cumulative effects of disease on indentured labourers in Mauritius during the late 1870s led the British Government to establish a Sanitary Commission in 1879.^119^ Its mandate was specifically geared towards understanding and arresting the spiralling effects of malaria. However, the 1878–79 outbreak of epidemic dropsy also featured. Charles Meldrum, a Mauritius-based meteorologist and astronomer, took a holistic, global, and long-term approach, associating heightened disease transmission to climatic anomalies, sunspots, and deforestation.^120^ He did so by identifying several ‘years of greatest mortality’ at about 11-year intervals in Mauritius across the nineteenth century and by linking them to similar temporal patterns in Ceylon (Sri Lanka), Australia, India, Gibraltar, Malta, Europe, and the Caribbean.^121^ In this context, he identified 1877–80 as global ‘years of greatest mortality’, with 1879 being the most severe in Mauritius, coinciding with the height of epidemic dropsy’s spread.^122^ Indeed, with 729 recorded deaths, he cited ‘*béri-béri* [sic.] or acute dropsy’ as the driver of heightened mortality in 1879.^123^ In so doing, he acknowledged the limits of contemporary colonial knowledge about what we now know to be epidemic dropsy in both India and Mauritius, while also inserting the disease into a global conversation about epidemics and death in the tropical world.

These developments were not lost on members of the colonial medical establishment in India. Although we now know that Meldrum’s ideas, which linked disease transmission to climate, environment, and their effects on miasmas, were misguided, they – and the broader Mauritian context they were situated in – contributed to a reframing of how some India-based medical officers understood the disease. For example, some theories that positioned epidemic dropsy as a ‘new disease’, which originated in late 1878, were given renewed vigour. One leading figure in this context was McLeod, who in May 1881 was the first to title a published article, ‘epidemic dropsy’, despite, at the time, suggesting questioningly that the disease could be a form of beriberi.^124^ In so doing, he used a term first deployed in Lovell’s report to describe the disease in Mauritius.^125^ Twelve years later, however, he built further on Lovell’s work by claiming that beriberi was ill-defined and arguing that the term would later ‘disappear from medical nomenclature’ as the ‘true pathology and causation’ of diseases to which this name had been attributed were discovered.^126^ Thus, in drawing on the medical literature from Mauritius, he challenged, increasingly directly, the majority position in colonial India’s medical establishment that understood epidemic dropsy in relation to beriberi.^127^ His interpretation of Lovell and others’ reports from Mauritius allowed him to distinguish between the Calcutta epidemic he witnessed and previous outbreaks of what was termed beriberi in other locales in India and Sri Lanka, including in the Madras Presidency in the late 1870s.

The core of the Indian colonial medical establishment was not to be cowed, however. Instead, they, like Meldrum in Mauritius, increasingly sought to interpret the 1877–80 epidemic through a global lens. They did so, however, to enhance the perceived importance of beriberi to medical scientific investigations and the role of colonial India’s medical establishment therein. In so doing, medical officers, such as Chevers, drew on medical reports in Mauritius to emphasise beriberi’s potentially epidemic characteristics and the possible international political, economic, and social ramifications that an outbreak could have. This marked a departure from the time of the epidemic itself, when the evidence suggests that acknowledging the possible epidemiological connection between India and Mauritius may have been seen by some as problematic.^128^ By contrast, Chevers wrote, ‘an analysis of the characteristics of the epidemic disease which first appeared in Calcutta in 1877, *and Mauritius in 1878*, enables us to declare… that acute beriberi is an exanthematous fever.’^129^ This use of evidence from Mauritius helped some in the colonial medical establishment in India to emphasise the importance of understanding beriberi as an infectious disease. Thus, according to Arnold, while before Chevers’ discussion of the 1877–80 epidemic ‘medical observers in India had… regarded beriberi as a local disease of secondary importance’, they understood it in global terms afterwards.^130^ Colonial medical practitioners in India used evidence from Mauritius to both discredit and emphasise the 1877–80 epidemic’s relevance to an understanding of beriberi.

## Conclusion

In conclusion, this article supports a trans-regional perspective for thinking about disease transmission and the circulation of colonial medical knowledge in the Indian Ocean World. Reconstruction of the first outbreak of epidemic dropsy recorded in documentary materials considering current medical knowledge shows how colonial economic and labour policies in India, Bengal, and Mauritius in the context of a variable global climate affected the likelihood of an epidemic occurring over a vast oceanic space. Such factors also affected different (groups of) people’s vulnerability to infection and mortality in these distant but interconnected locales. Contrary to most existing studies of disease transmission across the Indian Ocean World, however, which regularly focus on diseases transmitted via human-to-human contact or through migration of vectors, this article explores the history of an epidemic spread via consumption of a contaminated foodstuff. This represents a new layer to our understanding of the Indian Ocean World as an inter-connected macro-region, including as a zone connected by disease transmission,^131^ which places further emphasis on the importance of histories of food production, availability, and circulation.

The measures that colonial governments took to limit the spread of epidemics between India, Bengal, and Mauritius show that there was also a contemporary acknowledgement of the trans-regional capacity of disease transmission in the Indian Ocean World. However, the evidence from the first recorded outbreak of epidemic dropsy shows that the ways in which colonial medical officers incorporated evidence and reports from other colonies could be highly contradictory. In India, initial attempts to understand the disease in Calcutta and wider colonial Bengal were limited by an inability to track the disease’s spread and by a pre-occupation with beriberi within its medical establishment. A more rigorous approach in Mauritius, meanwhile, which was enabled by its colonial physicians drawing a direct linkage between advancements in medical science and the health of the colonial economy, contributed to both a challenge and a reinforcement of this pre-occupation: some medical officers, notably McLeod, drew on the Mauritius-based medical literature to question scientific investigations about beriberi in India to that point; others, such as Chevers, used the evidence from Mauritius to stress the urgency of investigating beriberi in India and globally moving forwards. Either way, the evidence supports recent trends in medical history that have stressed the multiple spatial and contextual origins of colonial medical knowledge.^132^

## Footnotes

1 See, for example: Anna Gerritsen and Burton Cleetus , ‘Health, Medicine, and Trade in the Indian Ocean World’, in Burton Cleetus and Anne Gerritsen (eds), Histories of Health and Materiality in the Indian Ocean World: Medicine, Material Culture and Trade, 1600-2000 (London: Bloomsbury, 2023), 1–24 ; Anna Winterbottom and Facil Tesfaye , ‘Introduction’, in Anna Winterbottom and Facile Tesfaye (eds), Histories of Medicine and Healing in the Indian Ocean World, Volume One (Basingstoke: Palgrave Macmillan, 2015), 1–35 ; Kapil Raj , ‘Beyond Postcolonialism and Postpositivism: Circulation and the Global History of Science’, Isis, 104, 2 (2013), 337–4723961692 10.1086/670951.

2 ENSO is an anomaly of sea surface temperatures in the eastern equatorial Pacific Ocean. El Niño refers to a positive anomaly, when sea surface temperatures are significantly above normal; La Nina refers to a negative anomaly, when sea surface temperatures are significantly below normal.

3 IOD is an anomaly of sea surface temperatures in the Indian Ocean. A positive IOD anomaly refers to when sea surface temperatures in the western half of the Indian Ocean are warmer than those of the eastern half; a negative IOD anomaly refers to the opposite phenomenon, wherein sea surface temperatures in the western half of the Indian Ocean are cooler than those of the eastern half.

4

Deepti Singh , Richard Seager , Benjamin I. Cook , Mark Cane , Mingfang Ting , Edward Cook , and Michael Davis , ‘Climate and the Global Famine of 1876-78’, Journal of Climate, 31, 23 (2018), 9445–67. This statement excepts the positive IOD anomaly (when ENSO was neutral) in 2019. See: Philip Gooding , ‘Introduction: Droughts, Floods, and Global Climatic Anomalies in the Indian Ocean World’, in Philip Gooding (ed.), Droughts, Floods, and Global Climatic Anomalies in the Indian Ocean World (Cham, CH: Palgrave-MacMillan, 2022), 1–3, 16–19.

5

Mike Davis , Late Victorian Holocausts: El Niño Famines and the Making of the Third World (London: Verso, 2001), 23–116 ; ZhiXin Hao , JingYun Zheng , GuoFeng Wu , ZueZhen Zhang , and QuanSheng Ge , ‘1876–78 Severe Drought in North China: Facts, Impacts and Climatic Background’, Chinese Science Bulletin, 55 (2010), 3001–7; David J. Nash , Kathleen Pribyl Jørgen Klein , Raphael Neukom , Georgina H. Endfield , George C.D. Adamson , and Dominic Kniveton , ‘Seasonal Rainfall Variability in Southeast Africa During the Nineteenth Century Reconstructed from Documentary Sources’, Climatic Change, 134 (2016), 610 ; David J. Nash , Kathleen Pribyl , Georgina H. Endfield , Jørgen Klein , and George C.D. Adamson , ‘Rainfall Variability Over Malawi During the Late Nineteenth Century’, International Journal of Climatology, 38, S1 (2018), 629–42; Fiona Williamson , ‘Responding to the Extremes: Managing Urban Water Scarcity in the Late Nineteenth Century Straits Settlements’, Water History, 12 (2020), 251–6333144897 10.1007/s12685-020-00260-6PMC7594933; Philip Gooding , ‘Tsetse Flies, ENSO, and Murder: The Church Missionary Society’s Failed Ox-Cart Experiment of 1876–78,’ Africa: Rivista semestrale di studi e ricerche, 1, 2 (2019), 21–36 ; Philip Gooding , ‘ENSO, IOD, Drought, and Floods in Equatorial Eastern Africa, 1876-1878’, in Gooding (ed.), Droughts, Floods, and Global Climatic Anomalies (Cham: Palgrave Macmillan, 2022), 259–87; Sunil Amrith , Unruly Waters: How Rains, Rivers, Coasts, and Seas Have Shaped Asia’s History (New York: Basic Books, 2018), 65–89 .

6 Davis, *Late Victorian Holocausts*, *op. cit.* (note 5), 25–59.

7 See, for example: *Ibid*; Gooding, ‘ENSO, IOD, Drought, and Floods,’ *op. cit.* (note 5), 277–78; Williamson, ‘Responding to Extremes,’ *op. cit.* (note 5), 254.

8 Thomas C. Peterson and Russell S. Vose, ‘An Overview of the Global Historical Climatology Network Temperature Database’, *Bulletin of the American Meteorological Society*, 78, 12 (1997), 2837–50. See below for malaria transmission.

9 Gooding, ‘Tsetse Flies, ENSO Peterson, and Murder’, *op. cit.* (note 5), 21–36.

10

Gwyn Campbell , Africa and the Indian Ocean World from Early Times to Circa 1900 (Cambridge: Cambridge University Press, 2019); David Arnold , ‘The Indian Ocean as Disease Zone, 1500-1950’, South Asia: Journal of South Asian Studies, 14, 2 (1991), 1–21 ; Eva-Maria Knoll and Gwyn Campbell , ‘Introduction’, in Gwyn Campbell and Eva-Maria Knoll (eds.), Disease Dispersion and Impact in the Indian Ocean World (Cham: Palgrave Macmillan, 2020), 1–23 .

11 Gerritsen Cleetus, ‘Health, Medicine, and Trade’, *op. cit.* (note 1), 1–24; Winterbottom and Tesfaye, ‘Introduction’, *op. cit.* (note 1), 1–35; Raj, ‘Beyond Postcolonialism and Postpositivism,’ *op. cit.* (note 1), 337–47.

12 For a summary, see: Projit Mukharji , Nationalizing the Body: The Medical Market, Print and Daktari Medicine (London: Anthem Press, 2009), 1–34 . But more recently: Anna Winterbottom , Hybrid Knowledge in the Early East India Company World (London: Palgrave Macmillan, 2016), 112–39; Projit Mukharji , ‘Parachemistries: Colonial Chemopolitics in a Zone of Contest’, History of Science, 54, 4 (2016), 362–8228027705 10.1177/0073275316681803; Projit Mukharji , ‘Swapnaushadhi: The Embedded Logic of Dreams and Medical Innovation in Bengal’, Culture, Medicine, and Psychiatry, 38 (2014), 387–407 24990459 10.1007/s11013-014-9387-6; Nabaparna Ghosh , A Hygienic City-Nation: Space, Community, and Everyday Life in Colonial Calcutta (Cambridge: Cambridge University Press, 2020), 1–24 .

13

B.D. Sharma , Sanjay Malhotra , Vikram Bhatia , and Mandeep Rathee , ‘Epidemic Dropsy in India’, Postgraduate Medical Journal, 75, 889, (1999), 657–6110621875 10.1136/pgmj.75.889.657PMC1741391.

14

Dr Chambers , ‘Notes on the Epidemic Fever and the New Disease (Acute Oedema)’, Indian Medical Gazette, 15, 4 (1880), 104 .

15 Sharma et al. ‘Epidemic Dropsy in India’, *op. cit.* (note 13), 657–61.

16

M.J. Shah , K.K. Manghani , U.K. Sheth , J.M. Mehta , and P.V. Karandikar , ‘Epidemic Dropsy: Epidemiological, Clinical, and Therapeutic Observations in 67 Cases’, Indian Journal of Medical Research, 57, 10 (1969), 1878–915372173 .

17 Sharma et al. ‘Epidemic Dropsy in India’, *op. cit.* (note 13), 657-61.

18

George Watt , A Dictionary of the Economic Products of India (Calcutta: Superintendent of Government Printing, 1885), 306–8.

19
*Ibid.*, 308.

20

Navneet Sharma , Naina Mohan , Ashish Bhalla , Aman Sharma , and Surjit Singh , ‘Changing Pattern of Epidemic Dropsy in North India’, Asia Pacific Journal of Medical Toxicology, 2 (2013), 87 .

21

David Arnold , ‘British India and the “Beriberi Problem”, 1798-1942’, Medical History, 54 (2010), 301 10.1017/s0025727300004622PMC288945620592882.

22

Norman Chevers , A Commentary on the Diseases of India (London: J and Churchill, 1886).

23 Cornish to Lovell, 4 August 1880, in *Indian Medical Gazette*, 17, 1 (1882), 26–7. Davis notes several famine-related diseases in the Madras Presidency at this time: Davis, *Late Victorian Holocausts*, *op. cit.* (note 5), 34.

24

Kenneth McLeod , ‘Epidemic Dropsy’, Transactions of the Epidemiological Society of London, 12 (1893), 89–90 PMC554252829419225; Rammay Ray , ‘Beri-beri’, Indian Medical Gazette, 15, 4 (1880), 89–91 PMC514432128998474; Rammay Ray , ‘Beri-beri’, Indian Medical Gazette, 15, 5 (1880), 119–22PMC514428728998450. McLeod’s article was also published in several instalments in the *Indian Medical Gazette* in 1893–4.

25 Watt, *A Dictionary of Economic Products*, *op. cit.* (note 18), 527, 529.

26 McLeod, ‘Epidemic Dropsy’, *op. cit.* (note 24), 75; J.F.P. McConnell, ‘The New Disease’, 14, 1 (1879), 25.

27 Alexander Springer, ‘A GIS Approach to a History of Epidemics in 19^th^ Century India’, *NiCHE* (2023): https://niche-canada.org/2023/03/15/a-gis-approach-to-a-history-of-epidemics-in-19th-century-india/ [Accessed: 21 June 2023]; Appraising Risk, ‘India, Disease, 1834-1910’ (2023): https://www.appraisingrisk.com/collected-data/ [Accessed: 21 June 2023]; Kenneth McLeod (see below) put the figure at 5,022 for these years, although it is unclear from which source he gathered this information: McLeod, ‘Epidemic Dropsy’, *op. cit.* (note 24), 76. These discrepancies point to the limitations of colonial disease statistics.

28 McLeod, ‘Epidemic Dropsy’, *op. cit.* (note 24), 60.

29 Investigations into later epidemics showed that the seasonal cycle and the varying availability of mustard seeds therein was crucial to the timing of outbreaks. See: S.B. Lal , ‘Presence of Argemone Mexicana Seeds in Mustard Seeds Grown in Bihar’, Indian Medical Gazette, 86, 7 (1951), 317–814880257 PMC5189242.

30 Watt, *A Dictionary of Economic Products*, *op. cit.* (note 18), 529.

31

William Wilson Hunter , A Statistical Account of Bengal, V (London: Trübner & Co, 1875), 308 .

32 McLeod, ‘Epidemic Dropsy’, *op. cit.* (note 24), 60–2; Joseph O’Brien ‘Acute Dropsy (Beriberi?)’, Indian Medical Gazette, 14, 1 (1879), 5–8 PMC514656128998734; Joseph O’Brien , ‘Is Acute Dropsy (The New Disease) Contagious?’, Indian Medical Gazette, 14, 5 (1879), 127–9PMC514647028998663; A. Crombie , ‘Acute Oedema in Dacca’, Indian Medical Gazette, 14, 3 (1879), 90 .

33 McLeod, ‘Epidemic Dropsy’, *op. cit.* (note 24), 75.

34 For beriberi, see: Arnold, ‘British India and the “Beriberi Problem”’, *op. cit.* (note 21), 295–314.

35 McLeod, ‘Epidemic Dropsy’, *op. cit.* (note 24), 76.

36

J. Worth Estes , ‘Dropsy’, in Kenneth F. Kiple (ed.), The Cambridge World History of Human Disease (Cambridge: Cambridge University Press, 1993), 689 .

37 McLeod, ‘Epidemic Dropsy’, *op. cit.* (note 24), 87; Ray, ‘Beri-beri’, *Indian Medical Gazette*, 15, 5 (1880), 120.

38 McLeod, ‘Epidemic Dropsy’, *op. cit.* (note 24), 76.

39
*Ibid.*, 72. Closer analysis of the police reports is contained in an earlier article written by McLeod’s: Kenneth McLeod , ‘Epidemic Dropsy in Calcutta’, Indian Medical Gazette, 16, 5 (1881), 148 PMC513871128997895.

40 McLeod, ‘Epidemic Dropsy’, *op. cit.* (note 24), 72.

41 See also: Chambers, ‘Notes on the Epidemic Fever’, *op. cit.* (note 14), 104. The author claimed, ‘There is not a day when I am not summoned to see one or more cases of the new fever [epidemic dropsy].’ This suggests it was wider spread than the police reports imply.

42 At least three contemporary reports suggest an awareness of this possibility. See: ‘Beri-beri in the Mauritius’, *Indian Medical Gazette*, 15, 8 (1880), 216; O’Brien, ‘Is Acute Dropsy… Contagious?’, *op. cit.* (note 32), 127; McConnell, ‘The New Disease’, *op. cit.* (note 26), 90.

43 For a wider discussion, see: Richard B. Allen, ‘Asian Indentured Labor in the 19^th^ and early 20^th^ Century Colonial Plantation World’, *Oxford Research Encyclopedia of Asian History* (2017): https://doi.org/10.1093/acrefore/9780190277727.013.33 [Accessed: 21 June 2023].

44

Allister MacMillan , Mauritius Illustrated: Historical and Descriptive Commercial and Industrial Facts, Figures, & Resources (New Delhi: Asian Educational Services, 2010 [1914]), 216 ; Dehoutee Vina Ballgobin , ‘Travailleurs engagés indiens et nutrition à l’île Maurice (1834-1900)’, Revue Historique de l’océan Indian (2013), 286 .

45 National Archives of Mauritius (NAM) B2 ‘Annual Report of the Protector of Immigrants for 1878’, 1; NAM B2 ‘Annual Report of the Protector of Immigrants for 1879,’ 2.

46

Raj Boodhoo , Health, Disease, and Indian Immigrants in Nineteenth Century Mauritius (Port Louis: Aapravasi Ghat Trust Fund, 2010); Yoshina Hurgobin , ‘Making Medical Ideologies: Indentured Labor in Mauritius’, in Anna Winterbottom and Facil Tesfaye (eds) Histories of Medicine and Healing in the Indian Ocean World, Volume Two (Basingstoke: Palgrave Macmillan, 2016), 1–26 ; Abdoo Cader Kalla , ‘The Inequalities of Morbidity and Mortality in Mauritius–Its Ethnic and Geographic Dimension’, Social Science & Medicine, 36, 10 (1993), 1276 10.1016/0277-9536(93)90217-r8511612.

47

Ashvin Nemchand , ‘The Recruitment and Voyage of Indentured Labourers from India’, in Aapravasi Ghat Trust Fund (ed.), Angaje: Explorations in the History, Society and Culture of Indentured Immigrants and the Descendants in Mauritius, I (Port Louis: Aapravasi Ghat Trust Fund, 2012), 124–5.

48
*Ibid.*, 123.

49 Indira Gyaram and Amirchandsingh Teerbhoohan, ‘Life at the Immigration Depot’, in Aapravasi Ghat Trust Fund (ed.), *Angaje*, II, 17. For a wider discussion on the multiple meanings and contexts of smallpox vaccination in the colonial world, see: Alison Bashford , Imperial Hygiene: A Critical History of Colonialism, Nationalism and Public Health (New York: Palgrave MacMillan, 2004), 14–38 .

50 See also: Marina Carter , Servants, Sirdars, and Settlers: Indians in Mauritius, 1834-1874 (Delhi: Oxford University Press, 1995), 9, 120–4.

51 NAM B2 ‘Annual Report of the Protector of Immigrants for 1879’, *op. cit.* (note 45), 36.

52 Although most depots for indentured labourers were located in Garden Reach, that which was for Mauritius was located in Bhowanipore. See: J.G. Garrow Grant , ‘Annual Report of the Protector of Emigrants,’ in Leela Gujadhur Sarup (eds.), Colonial Emigration 19th-20th Century: Annual Reports From The Port Of Calcutta To British And Foreign Colonies (Kolkata: Aldrich International, 2008), I, 259 .

53

Ashutosh Kumar , Coolies of the Empire: Indentured Indians in the Sugar Colonies, 1830-1920 (Cambridge: Cambridge University Press, 2007), 107 ; Huguette Ly-Tio-Fane Peneo , Lured Away: The Life History of Indian Cane Workers in Mauritius (Moka, Mauritius: Mahatma Gandhi Institute, 2009), 110 .

54 Clarenc to Lovell, 22 September 1880, *Indian Medical Gazette*, 17, 1 (1882), 26.

55

Francis Lovell , ‘Report on Acute Anaemic Dropsy in Mauritius’, Indian Medical Gazette, 17, 1 (1882), 26 PMC515181028998792; Andrew Davidson , ‘Acute Anaemic Dropsy; an Epidemic Disease recently observed in Mauritius and India’, Edinburgh Medical Journal, 27, 2 (1881), 123–4.

56 NAM B2 ‘Annual Report of the Protector of Immigrants for 1879,’ *op. cit.* (note 45), 36.

57 This conclusion contradicts the inference of Andrew Davidson, who investigated the outbreak in Mauritius in the late 1870s and early 1880s: Davidson, ‘Acute Anaemic Dropsy’, *op. cit.* (note 55), 118–9.

58 Gyaram and Teerbhoohan, ‘Life at the Immigration Depot’, *op. cit.* (note 49), 4; Kader A. Parahoo , ‘Early Colonial Health Developments in Mauritius’, International Journal of Social Determinants of Health and Health Services, 16, 3 (1986), 418 10.2190/NNNL-WT10-QPTM-AWHJ3525435.

59

Marina Carter , ‘Strategies of Labour Mobilisation in Colonial India: The Recruitment of Indentured Workers for Mauritius’, Journal of Peasant Studies, 19, 3–4 (1992), 230 ; Richard B. Allen , Slaves, Freedmen, and Indentured Laborers in Colonial Mauritius (Cambridge: Cambridge University Press, 1999), 114–69.

60 Nagamah Gopauloo, ‘Fruit and Vegetable Sellers in the Port Louis Market’, in Aapravasi Ghat Trust Fund, *Angaje*, II, 185.

61

John Gilbert Baker , Flora of Mauritius and the Seychelles: A Description of the Flowering Plants and Ferns of those Islands (London: L. Reeve, 1877).

62 Carter, *Servants, Sirdars, and Settlers*, *op. cit.* (note 50), 165–7.

63 See also: Ballgobin, ‘Travailleurs engagés’, *op. cit.* (note 44), 297; Carter, *Servants, Sirdars, and Settlers*, *op. cit.* (note 50), 167.

64 Davidson ‘Acute Anaemic Dropsy’, *op. cit.* (note 55), 118.

65 McLeod, ‘Epidemic Dropsy’, *op. cit.* (note 24), 62.

66 Davidson, ‘Acute Anaemic Dropsy’, *op. cit.* (note 55), 127; McLeod, ‘Epidemic Dropsy’, *op. cit.* (note 24), 62.

67 McLeod, ‘Epidemic Dropsy’, *op. cit.* (note 24), 62.

68
*Ibid.*

69 Boodhoo, *Health, Disease, and Indian Immigrants*, *op. cit.* (note 46), 191.

70 Davidson ‘Acute Anaemic Dropsy’, *op. cit.* (note 55), 118.

71 Nemchand, ‘The Recruitment and Voyage of Indentured Labourers’, *op. cit.* (note 47), 137.

72 McLeod, ‘Epidemic Dropsy’, *op. cit.* (note 24), 62.

73
*Ibid.*; ‘Beri-beri in the Mauritius’, *op. cit.* (note 42), 216; Francis Lovell, ‘Report on Acute Anaemic Dropsy in Mauritius’, *Indian Medical Gazette*, 16, 12 (1881), 344; NAM B2 ‘Annual Report of The Protector of Immigrants for 1879’, *op. cit.* (note 45), 12.

74 McLeod, ‘Epidemic Dropsy’, *op. cit.* (note 24), 62.

75 ‘Beri-beri in the Mauritius’, *op. cit.* (note 42), 216; Clarenc to Lovell, 22 September 1880, 26; Davidson, ‘Acute Anaemic Dropsy’, *op. cit.* (note 55), 118–9.

76 NAM B2 ‘Immigration of 1859. Report Thereupon by the Protector of Immigrants,’ 1.

77 Gopauloo, ‘Fruit and Vegetable Sellers’, *op. cit.* (note 60), 185; Allen, *Slaves, Freedmen, and Indentured Laborers*, *op. cit.* (note 59), 162.

78 Carter, ‘Strategies of Labour Mobilisation’, *op. cit.* (note 59), 233–4.

79 Allen, *Slaves, Freedmen, and Indentured Laborers*, *op. cit.* (note 59), 162.

80

K. Hazareesingh , ‘The Religion and Culture of Indian Immigrants in Mauritius and the Effects of Social Change’, Comparative Studies in Society and History, 8, 2 (1966), 248 .

81 Ballgobin, ‘Traveilleurs engagés’, *op. cit.* (note 44), 287.

82 For more on divergent gender roles in Mauritius during the period of indenture, see: Allen, *Slaves, Freedmen, and Indentured Laborers*, *op. cit.* (note 59), 162–3; Ramola Ramtohul, ‘Women in Mauritius’, *Oxford Research Encyclopedia of African History* (2020): https://doi.org/10.1093/acrefore/9780190277734.013.528 [Accessed: 24 July 2023]; Marina Carter, *Lakshmi’s Legacy: The Testimonies of Indian Women in 19^th^-Century Mauritius* (Rose-Hill, Mauritius: Editions de l’Ocean Indien, 1994).

83 Ballgobin, ‘Traveilleurs engagés’, *op. cit.* (note 44), 297. This was also hypothesised by colonial medical officers: Lovell, ‘Report on Acute Anaemic Dropsy’, *op. cit.* (note 73), 346.

84 Chambers, ‘Notes on the Epidemic Fever and the New Disease’, *op. cit.* (note 14), 104.

85 McCleod, ‘Epidemic Dropsy’, *op. cit.* (note 24), 55–93.

86 Gerritsen and Cleetus, ‘Health, Medicine, and Trade’, 1–24; Winterbottom and Tesfaye, ‘Introduction’, *op. cit.* (note 1), 1–35; Raj, ‘Beyond Postcolonialism and Postpositivism’, *op. cit.* (note 1), 337–47.

87

Michael Christopher Low , Imperial Mecca: Ottoman Arabia and the Indian Ocean Hajj (New York: Columbia University Press, 2020), 139–40; Mark Harrison , Climates & Constitutions: Health, Race, Environment, and British Imperialism in India, 1600-1850 (New Delhi: Oxford University Press, 2002); Suman Seth , Difference and Disease: Medicine, Race, and the Eighteenth-Century British Empire (Cambridge: Cambridge University Press, 2018); Carter, *Servants, Sirdars, and Settlers*, *op. cit.* (note 50), 124.

88 For a summary, see: McLeod, ‘Epidemic Dropsy’, *op. cit.* (note 24), 73–8.

89 Davidson, ‘Acute Anaemic Dropsy’, *op. cit.* (note 55), 124–5.

90 Arnold, ‘British India and the “Beriberi Problem”’, *op. cit.* (note 21), 311–2.

91

A. Deva Sagayam , ‘An Epidemic of Dropsy among Indians in Fiji’, Indian Medical Gazette, 62, 9 (1927), 506–729010756 PMC5197852.

92 McConnell, ‘The New Disease,’ *op. cit.* (note 26), 90; O’Brien, ‘Is Acute Dropsy… Contagious?’, *op. cit.* (note 32), 127–9.

93 For the state of colonial medical knowledge related to beriberi in the nineteenth-twentieth centuries, including in the 1870s–80s, see: Arnold, ‘British India and the “Beriberi Problem”’, *op. cit.* (note 21), 295–314.

94 O’Brien, ‘Acute Dropsy’, *op. cit.* (note 32), 5–8; ‘A New Disease’, *Indian Medical Gazette*, 13, 10 (1878), 287–8; ‘Editorial’, *Indian Medical Gazette*, 14, 1 (1879), 22–3.

95 Ray, ‘Beri-Beri’, *Indian Medical Gazette*, 15, 4 (1880), 89–91; Ray, ‘Beri-beri’, *Indian Medical Gazette*, 15, 5 (1880), 119–22.

96 McLeod, ‘Epidemic Dropsy in Calcutta’, *op. cit.* (note 39), 149.

97

J.F. Evans , ‘A Note on the Pathology of Kala-Azar or Beri-Beri of Assam’, Indian Medical Gazette, 27, 11 (1892), 330–2PMC513709129000094; Mary E. Gibson , ‘The Identification of Kala Azar and the Discovery of *Leishmania Donovani*
’, Medical History, 27, 2 (1983), 208 10.1017/s0025727300042691PMC11393086345968.

98 Arnold, ‘British India and the “Beriberi Problem”’, *op. cit.* (note 21), 302.

99 Ray, ‘Beri-beri’, *Indian Medical Gazette*, 15, 5 (1880), 120; McLeod, ‘Epidemic Dropsy’, *op. cit.* (note 24), 87.

100 Arnold, ‘British India and the “Beriberi Problem”’, *op. cit.* (note 21), 302. See also: Mark Harrison , Medicine in an Age of Commerce and Empire: Britain and its Tropical Colonies 1660-1830 (Oxford: Oxford University Press, 2010), 1–25 .

101 ‘Beri-beri in the Mauritius’, *op. cit.* (note 42), 216–7.

102 Ray, ‘Beri-beri’, *Indian Medical Gazette*, 15, 5 (1880), 120; McLeod, ‘Epidemic Dropsy’, *op. cit.* (note 24), 87.

103 Low, *Imperial Mecca*, *op. cit.* (note 87), 141.

104 ‘The Calcutta Medical Society’, *Indian Medical Gazette*, 15, 8 (1880), 221–2.

105 Lovell, ‘Report on Acute Anaemic Dropsy’, *op. cit.* (note 73); Lovell, ‘Report on Acute Anaemic Dropsy’, *op. cit.* (note 55).

106 Lovell, ‘Report on Acute Anaemic Dropsy’, *op. cit.* (note 73), 344.

107
*Ibid.*, 348. Davidson repeated this characterisation in his report in the *Edinburgh Medical Journal.* Davidson, ‘Acute Anaemic Dropsy’, *op. cit.* (note 55), 128.

108 ‘Beri-beri in the Mauritius’, *op. cit.* (note 42), 216.

109 Lovell, ‘Report on Acute Anaemic Dropsy’, *op. cit.* (note 73), 346–7.

110 NAM B2 ‘Annual Report of the Protector of Immigrants for 1879’, *op. cit.* (note 45), 36. Lovell did not even refer to the report of the Depot Medical Officer in his writing about ‘acute anaemic dropsy’. In fact, he was scathing of the Immigration Department as a whole for being unable to provide him with data about the distribution of new arrivals to different estates, which may have aided his investigation: Lovell, ‘Report on Acute Anaemic Dropsy’, *op. cit.* (note 55), 26.

111 Lovell, ‘Report on Acute Anaemic Dropsy’, *op. cit.* (note 73), 342–8.

112 Hurgobin, ‘Making Medical Ideologies’, *op. cit.* (note 46), 4.

113 See also: Carter, *Servants, Sirdars, and Settlers*, *op. cit.* (note 50), 124. This was a condition that continued into subsequent decades. See: J.W.P. Muir-Mackenzie, *The End of Indentured Immigration: 1895 Report on the Condition of Indian Immigrants in Mauritius by J.W.P. Mackenzie: The Distribution list of the SS Surada, the Last Ship Carrying Labourers, 1923* (Mauritius: University of Mauritius, Centre for Research on Slavery and Indenture, 2017), 16.

114
*Ibid.*, 14; Parahoo, ‘Early Colonial Health Developments in Mauritius’, *op. cit.* (note 58), 416.

115 Parahoo, ‘Early Colonial Health Developments in Mauritius,’ *op. cit.* (note 58), 416.

116 For wider discussion, see: Rohan Deb Roy , Malarial Subjects: Empire, Medicine and Nonhumans in British India, 1820-1909 (Cambridge: Cambridge University Press, 2017), 106–729400918.

117 See, for example: Philip D. Rotz , ‘Sweetness and Fever? Sugar Production, *Aedes aegypti*, and Dengue Fever in Natal, South Africa, 1926–1927’, South African Historical Journal, 68, 3 (2016), 288 .

118 Peterson and Vose, ‘An Overview of the Global Historical Climatology Network’, *op. cit.* (note 8), 2837–50. Many of the effects of the 1877 anomalies in the wider world are discussed above. The effects of the 1867 global climatic event are less well-known, though they have received attention in the context of typhoon histories in the Philippines. See: James Francis Warren, ‘The Great Ilocos Flood of 1867’, in Gooding (ed.), *Droughts, Floods, and Global Climatic Anomalies*, *op. cit.* (note 4), 199–229.

119 NAM B1B Report of the Sanitary Commission – 1882 (SC – 1882): Mauritius. Report of the Sanitary Commission appointed to Enquire into the Health Laws of the Colony (1882).

120

Charles Meldrum , Weather, Health, and Forests: A Report on the Inequalities of the Mortality from Malarial Fever and Other Diseases, in Mauritius, Considered in Relation to the Inequalities of Temperature, Humidity, and Rainfall; on a Possible Periodicity of Mortality Related to the Eleven-Year Periodicity of Solar Activity; and on the Climatic and Other Effects of Forests / Prepared for the Sanitary Commission of Mauritius appointed by Proclamation No. 50 of 1879 (Mauritius: Mercantile Record Company Printing Establishment, 1881).

121
*Ibid.*, 152.

122
*Ibid.*, 3.

123
*Ibid.*, 29.

124 McLeod, ‘Epidemic Dropsy in Calcutta’, *op. cit.* (note 39), 149.

125 ‘The Calcutta Medical Society’, *op. cit.* (note 104), 221; Lovell, ‘Report on Acute Anaemic Dropsy’, *op. cit.* (note 73), 344.

126 McLeod, ‘Epidemic Dropsy’, *op. cit.* (note 24), 92.

127 See his criticism of Chevers, for example: *Ibid.*, 91.

128 Low, *Imperial Mecca*, *op. cit.* (note 87), 191.

129 Chevers, *A Commentary on the Disease of India*, *op. cit.* (note 22), 62. Emphasis added.

130 Arnold, ‘British India and the “Beriberi Problem”’, *op. cit.* (note 21), 302.

131 Arnold, ‘The Indian Ocean as Disease Zone’, 1–21; Knoll and Campbell, ‘Introduction’, *op. cit.* (note 10), 1–23.

132 Gerritsen and Cleetus, ‘Health, Medicine, and Trade,’ *op. cit.* (note 1), 1–24; Winterbottom and Tesfaye, ‘Introduction’, *op. cit.* (note 1), 1–35; Raj, ‘Beyond Postcolonialism and Postpositivism’, *op. cit.* (note 1), 337–47.

